# Relationship between *HLA‐DPA1* genetic polymorphism and anembryonic pregnancy

**DOI:** 10.1002/mgg3.1046

**Published:** 2019-11-30

**Authors:** Zhendong Wang, Xiaolin Lu, Xiuying Yao, Xinli Liu, Linlin Zhao, Shaoyan Chang, Ting Zhang, Bo Niu, Li Wang

**Affiliations:** ^1^ Laboratory of Biochemistry and Molecular Biology Shanxi Medical University Shanxi China; ^2^ Beijing Municipal Key Laboratory of Child Development and Nutriomics Capital Institute of Pediatrics Beijing P.R. China; ^3^ Department of Obstetrics and Gynecology PLA General Hospital 263th Clinical Department Beijing China

**Keywords:** anembryonic pregnancy, association study, *HLA‐DPA1*, Immune Factors, Single‐nucleotide polymorphism (SNP)

## Abstract

**Background:**

Human leukocyte antigen (HLA)‐DP is an HLA class II molecule. Overexpression of HLA class II molecules in placental trophoblast cells may induce pregnancy loss. However, the association between HLA‐DP and pregnancy loss remains unclear. HLA‐DPA1 is an HLA‐DP peptide chain. The objective of this study was to assess the association between *HLA‐DPA1* genetic polymorphism and anembryonic pregnancy, a type of early pregnancy loss, in the Chinese population.

**Methods:**

A case–control study was designed to compare the frequencies of *HLA‐DPA1* gene polymorphisms in an anembryonic pregnancy group and a control group. Sixty‐eight cases and 122 controls were recruited. Statistical analysis was performed to assess the correlation between single‐nucleotide polymorphisms (SNPs) and anembryonic pregnancy susceptibility. MassARRAY high‐throughput DNA analysis was used to analyze 19 *HLA‐DPA1* SNPs. To explore how *HLA‐DPA1* polymorphism could affect anembryonic pregnancy, HLA‐DPA1 serum levels were analyzed by ELISA.

**Results:**

Homozygous typing of rs1431403 (CC and TT) significantly increased the risk of anembryonic pregnancy in the case group (OR_CC_ = 3.13, 95% CI: 1.50–6.53; OR_TT_ = 2.96, 95% CI: 1.31–6.66; OR_CC+TT_ = 3.06, 95% CI: 1.62–5.78). In samples with high HLA‐DPA1 levels (≥1,500 pg/ml), the homozygous rs1431403 genotypes (n_CC_ = 21, 43.8%; n_TT_ = 20, 57.1%) were observed more frequently than were heterozygous genotypes.

**Conclusion:**

*HLA‐DPA1* rs1431403 may be a risk factor for anembryonic pregnancy in the Chinese population. Homozygous rs1431403 genotypes (CC and TT) may increase the risk of anembryonic pregnancy by aberrantly increasing the HLA‐DPA1 levels.

## Graphical abstract

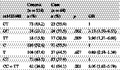

## 1 BACKGROUND

Anembryonic pregnancy, also known as blighted ovum, signifies that the embryo never developed or was reabsorbed, leading to pregnancy loss (Chaudhry & Siccardi, 2019; Nguyen & Nickels, 2016; Sajan, Pulikkathodi, Vahab, Kunjitty, & Imrana, 2015). Anembryonic pregnancy is a common first trimester pregnancy loss and accounts for 50% of all spontaneous abortions (Chaudhry & Siccardi, 2019). Women with anembryonic pregnancies usually miscarry before 12 weeks, and anembryonic pregnancies are a common first‐trimester pregnancy loss in China.

Immune factors are closely related to embryo development. In a fetus, half of the genetic material is provided by the male parent. This makes the fetus equivalent to a semi‐allograft, which may cause rejection by the maternal immune system (Nakashima, Shima, Inada, Ito, & Saito, 2012). Previous studies found that embryonic implantation involves the complicated immune tolerance of the matrix‐fetus, and that immune system regulation plays an important role in maintaining immune tolerance toward the fetus, and inhibiting maternal immune system attack during the gestation period (Mellor & Munn, 2000; Warning, Mccracken, & Morris, 2011).

Human leukocyte antigen (HLA), also known as the major histocompatibility complex, is responsible for antigen presentation in the immune system and is generally divided into three categories I, II, and III (Mellor & Munn, 2000; Zhao, Sher, & Peters, 2018). Studies have confirmed that genetic polymorphism of HLA class I molecules is related to miscarriage. There are three subtypes of HLA class I molecules expressed in human placental trophoblast, HLA‐C, G, and E (Juch, Blaschitz, Dohr, & Hutter, 2012). Berger et al. found that the distribution of 12 single‐nucleotide polymorphisms (SNPs) in the promoter region of HLA‐G significantly differed between pregnancy loss and control groups (Berger, Hogge, Barmada, & Ferrell, 2010). Agrawal et al. found that HLA‐G polymorphism increased the risk of idiopathic recurrent spontaneous abortion (RSA) (Agrawal, Prakash, Misra, Phadke, & Agrawal, 2015).

Less attention has been paid to the association between HLA class II molecules and pregnancy loss than has been paid to HLA class I molecules. HLA class II molecules are heterodimeric, composed of an alpha (A1) and a beta chain (B1), and anchored in the cell membrane. It is generally believed that the function of HLA class II molecules is to bind exogenous antigenic peptides outside cells so that they can be recognized by CD4 + T cells. In placental trophoblasts, HLA II antigen suppression is an important mechanism for successful pregnancy. The overexpression of HLA class II molecules in placental trophoblastic cells may induce pregnancy loss. Inhibition of HLA class II gene expression in placental trophoblastic cells is important to pregnancy outcomes, and class II gene expression is observed in certain placental inflammatory areas associated with RSA (Kolte, Steffensen, Christiansen, & Nielsen, 2016). There are six subtypes of HLA class II molecules, HLA‐DP, ‐DQ, ‐DR, ‐DN, ‐DO, and ‐DM. HLA‐DP, ‐DQ, and ‐DR are classical HLA class II molecules and have attracted wide attention. Patients with RSA have higher HLA‐DQ or HLA‐DR frequency (D'Ippolito et al., 2016; Shimada, Ebina, Iijima, Deguchi, & Yamada, 2017). However, the relationship between HLA‐DP and pregnancy loss has not been addressed. An early study indicated that the role of HLA‐DPB1 in pregnancy loss was unknown, and there was a lack of sufficient evidence to prove that HLA‐DP plays a role of in pregnancy loss (Sagot et al., 1995). However, the role of HLA‐DPA1 (OMIM 142880), the HLA‐DP alpha peptide chain, has never been researched in pregnancy loss.

Given the vital role of HLA class II molecules in immune tolerance during pregnancy, we designed this study to analyze the association between *HLA‐DPA1* genetic polymorphisms and anembryonic pregnancy, and to explore whether aberrant HLA‐DPA1 expression induces anembryonic pregnancy.

## 2 MATERIALS AND METHODS

### 2.1 Ethical compliance

All participants submitted informed consent, and the study protocol was reviewed and approved by the local ethics committee and institute review board of the Capital Institute of Pediatrics.

### 2.2 Study design

A case–control study was designed to determine if there is an association between *HLA‐DPA1* (GRCh37.p13) SNPs and anembryonic pregnancy in the Chinese population. Pregnant women who were receiving prenatal care or had delivered between May 2016 and December 2017 were recruited from No. 263 Clinic of PLA Army General Hospital. Our surveillance projects were performed following B‐mode ultrasound screen‐based diagnosis to identify the anembryonic pregnancy and normal pregnancy groups. According to the guidelines from the American college of Obstetricians and Gynecologists, anembryonic pregnancy was diagnosed for the following: crown–rump length of 7 mm or greater and no heartbeat, mean sac diameter of 25 mm or greater and no embryo, the absence of embryo with heartbeat 2 weeks or more after a scan that showed a gestational sac without a yolk sac, or the absence of embryo with heartbeat 11 days or more after a scan that showed a gestational sac with a yolk sac (Sarah, Vanessa, & Rebecca, 2018). The diagnosis was confirmed during ultrasound examination 1 week later. Control samples were aborted from patients with normal pregnancies who chose to have an elective abortion. All participants had no special disease, were not taking any other drugs, and had no prior radiation effects. Chromosomal causes of spontaneous and nonspontaneous abortion were excluded by chromosome number variation detection (data not shown). A total of 190 individuals were recruited for this study, including 122 controls and 68 subjects with anembryonic pregnancy. Following the submission of informed consent, the medical staff collected clinical information, including gestational age (in weeks) and peripheral blood samples. The subjects were all of Han nationality.

### 2.3 DNA extraction

All samples were stored at −30°C in the hospital before being transported to the laboratory on ice. Genomic DNA was extracted from frozen blood samples using a blood and cell culture DNA kit (Qiagen), following the manufacturer's instructions. The concentration and purity of DNA were determined by absorbance at 260 and 280 nm using a NANODROP 1,000 Spectro photometer (Thermo SCIENTIFIC).

### 2.4 SNP selection and genotyping

Tag SNPs in the promoter and full‐length *HLA‐DPA1* gene regions were selected from the 1,000 Genomes database. Exonic SNPs and hot SNPs were obtained from the NCBI SNP database and NCBI PubMed database, respectively. Linkage correlation of the above SNPs was analyzed by Haploview 4.2 software (https://www.softpedia.com/get/Science-CAD/Haploview.shtml). Primers were designed using the AgenaCx website (https://agenacx.com/) for SNPs with a minor allele frequency greater than 0.10 in the Han population and strong linkage disequilibrium with other SNPs (*n* = 19), r2 was set to below 0.8. Polymerase chain reaction (PCR) was used to amplify the polymorphic regions. Genotyping was conducted by an experienced technician who was blinded to the diagnosis. We used a MassARRAY high‐throughput DNA analyzer with matrix‐assisted laser desorption/ionization time‐of‐fight mass spectrometry (Agena). To confirm the genotyping results, the negative controls were set and 60% of samples were regenotyped. Analyses of genotype/allele frequency were conducted for 14 *HLA‐DPA1* gene SNPs with genotyping call rates ≥90% (Table 1). Primers were synthesized by Sangon Biotech. The SNPs genotype and allele frequencies in other populations were acquired from the 1,000 Genomes database.

**Table 1:** Details of SNPs and primers used

Rank	SNP	pHWE*	MAF	Wild type	Allele	Location	Forward primer	Reverse primer	Genotype ratio
1	rs1042153	0.608	0.310	G	A/G	exon	ACGTTGGATGCGCCCAGCTCGTAGTTGTG	ACGTTGGATGGGAACAGCCAGAAGGACATC	92.11
2	rs1431403	0.134	0.458	T	C/T	intron	ACGTTGGATGAGTGACACGGCACATGTTTG	ACGTTGGATGCACAGCACCTTAATTTCCCC	93.68
3	rs2071349	0.323	0.160	C	C/G	intron	ACGTTGGATGCTTCAGAGCAAAGAAAACGC	ACGTTGGATGTGCACTTAAGATGACGGAGG	98.95
4	rs2073522	0.000	0.228	A	A/G	intron	ACGTTGGATGACATGACAGCTCTGCCTATG	ACGTTGGATGTGACACTGAACAGTGTCAGG	98.95
5	rs2308927	0.347	0.171	G	A/G	exon	ACGTTGGATGTCAGCGACACCCTCAGTGAC	ACGTTGGATGCCTCATCTGCCACATTGACA	94.74
6	rs2567279	0.900	0.273	T	C/T	intron	ACGTTGGATGATGGGAGAATCCACACTCAG	ACGTTGGATGAGCTCCTTGAGTCCAGATTC	96.84
7	rs3077	1.000	0.439	A	A/G	exon	ACGTTGGATGGAAGGGTCAGCAATTCAGTC	ACGTTGGATGCTGCCCTACAAACTCCATCT	100
8	rs3135022	0.167	0.283	A	A/G	intron	ACGTTGGATGTGATGCTGGGCAGTGAAAGG	ACGTTGGATGTCTCATTCCCCCTACAAACC	96.32
9	rs7770418	0.874	0.152	T	C/T	intron	ACGTTGGATGAAGTTCAAGTTCTCCTCCCC	ACGTTGGATGAATCTCCTGAATACAGCCCC	99.47
10	rs9277341	0.810	0.401	C	C/T	intron	ACGTTGGATGTCTTCTAAGTGCAATAGCCC	ACGTTGGATGCAAAAGCCACTTCTCTCAGG	98.42
11	rs9277348	0.523	0.114	T	C/T	exon	ACGTTGGATGAACTCCCCCACGTCGCTGTC	ACGTTGGATGAACTCCCCCACGTCGCTGTC	98.95
12	rs9277359	0.866	0.459	C	A/C	exon	ACGTTGGATGAACGCAATTGGCAAAAACTG	ACGTTGGATGTCTGGCAGTTGATTACTGTG	98.95
13	rs9378176	0.836	0.091	A	A/G	exon	ACGTTGGATGTCCACTGTGCTGCTGAGGT	ACGTTGGATGAAACCCAAGTGCAGTGTGAG	92.11
14	rs987870	0.547	0.235	A	A/G	Intron	ACGTTGGATGAAGTGTCTAAAGTAAGAAG	ACGTTGGATGCCACCTCCTTCAAAGCATCT	96.84
15	rs2301227	0.018	0.261	A	A/G	intron	ACGTTGGATGCTGGGACTAGGGTGATGTAG	ACGTTGGATGGAGTTCTTTTGGTATCCCCC	76.84
16	rs3135021	0.273	0.345	G	A/G	intron	ACGTTGGATGTTACACCCTTCCTCCTAGAC	ACGTTGGATGCCTTGCTACCCTGAAAGATG	85.79
17	rs6457711	0.064	0.492	A	A/C	intron	ACGTTGGATGCTAGTTCTAGATCTTTGAGG	ACGTTGGATGTCGCCATTTTAACTGGTGTG	80.53
18	rs66951571	1.000	0.268	G	G/T	intron	ACGTTGGATGAAGAGCACATAGGAGCCAAG	ACGTTGGATGTGGAGACATGAAGCATGTGG	7.37
19	rs7770501	1.000	0.290	C	C/G	exon	ACGTTGGATGCCTTTTTTCACCCCACATGC	ACGTTGGATGAGCATGGAGTGAGGAGGAC	4.74

Abbreviations: MAF, Minor allele frequency.

* *p* value for deviation from Hardy–Weinberg equilibrium.

### 2.5 Determination of HLA‐DPA1 protein levels

Serum HLA‐DPA1 levels were measured using the Human HLA‐DPA1 ELISA Kit (Jining Shiye), following the manufacturer's instruction manual. The HLA‐DPA1 concentration was calculated using GraphPad Prism (version 7). The logarithm of concentration was adopted to ensure the accuracy of statistical analyses.

### 2.6 Statistical analysis

Statistical analysis was performed using Statistical Package for the Social Science (SPSS, version 17.0). All tests were two‐sided, and *p* < .05 was considered significant. Chi‐square tests were conducted to evaluate the Hardy–Weinberg equilibrium (HWE) and to analyze the relationship between HLA‐DPA1 level and SNP genotype. Adjusted OR values with 95% confidence interval (CI) were performed by multiple logistic regression to judge the risk of anembryonic pregnancy related to the *HLA‐DPA1* polymorphism. Gestational weeks and HLA‐DPA1 expression were compared between case and control groups using independent sample *t* tests. The distribution of HLA‐DPA1 protein level was positively skewed, so the raw data were transformed logarithmically. This study had 85% power to detect an odds ratio (OR) of 2.55 at the 5% significance level based on the smallest sample sizes with 68 cases and 122 controls, which was calculated by Quanto software (http://biostats.usc.edu/Quanto.html).

### 2.7 Binding factor predictions

SNP binding factors were predicted by Alibaba2.1 (http://gene-regulation.com/pub/programs/alibaba2/index.html).

## 3 RESULTS

### 3.1 Genotype distribution of HLA‐DPA1 polymorphisms

All analyzed SNPs conformed to HWE with the exception of rs2073522, which deviated from HWE (*p* < .05). Statistical analysis was performed for 13 *HLA‐DPA1* SNP sites, 90% of which were successfully genotyped in our populations. Comparison of SNP frequencies among groups revealed very similar genotype and phenotype distributions for all polymorphisms with the exception of rs1431403 (data not shown).

The genotyping call rate for rs1431403 was 93.68% in 114 controls and 64 cases. The mean gestational week of all subjects was 7 weeks, and there were no significant differences in gestational week and maternal age between the case and the control groups (Table 2).

**Table 2:** Characteristics of samples in the case and control groups

Characteristic	Control (*N* = 114)	Case (*N* = 64)	*p* *
Age^a^	27.48 ± 6.59	29.16 ± 6.74	.109
Gestational week^a^	7.11 ± 1.20	7.45 ± 1.56	.099

^a^ Values were mean ± *SD*.

* Student's *t* test was used to calculate the *p* values.

### 3.2 Genotypes of rs1431403 in control and case groups

Of the 64 case group samples, 24 and 17 were rs1431403 CC and TT, respectively, and 23 were CT. Of the 114 control samples, 24, 18, and 72 were rs1431403 CC, TT, and CT, respectively. The proportion of rs1431403 homozygotes was significantly higher in the case group than in the control group, while the proportion of the CT genotype was similar in both groups. Although no significant difference in allele frequency was found between the control and case groups (*p* > .05), homozygous genotypes were significantly increased anembryonic pregnancies (OR_CC_ = 3.13, 95% CI: 1.50–6.53, *p* = .002; OR_TT_ = 2.96, 95% CI: 1.31–6.66, *p* = .009). Additionally, the proportion of case samples with the homozygous mutation combination (CC + TT) in the case group was significantly higher than that in the control group (OR = 3.06, 95% CI: 1.62–5.78, *p* = .001) (Table 3). These results suggest that the rs1431403 polymorphism may be positively associated with the risk of anembryonic pregnancy in this population.

**Table 3:** Genotypes of rs1431403 in control and case groups

rs1431403	Control (*n* = 114) *n* (%)	Case (*n* = 64) *n* (%)	*p*	OR
CT	72 (63.2)	23 (35.9)		1
CC	24 (21.1)	24 (37.5)	.002	3.13 (1.50–6.53)
TT	18 (15.8)	17 (26.6)	.009	2.96 (1.31–6.66)
C	120 (52.6)	71 (55.5)		1
T	108 (47.4)	57 (44.5)	.607	0.89 (0.58–1.38)
CT	72 (63.2)	23 (35.9)		1
CC + TT	42 (36.8)	41 (64.1)	.001	3.06 (1.62–5.78)

Note

Chi‐squared tests were used to calculate *p* values. Fisher's exact test was used when the sample size was less than 5.

### 3.3 HLA‐DPA1 protein expression in control and case groups

**Figure 1:**
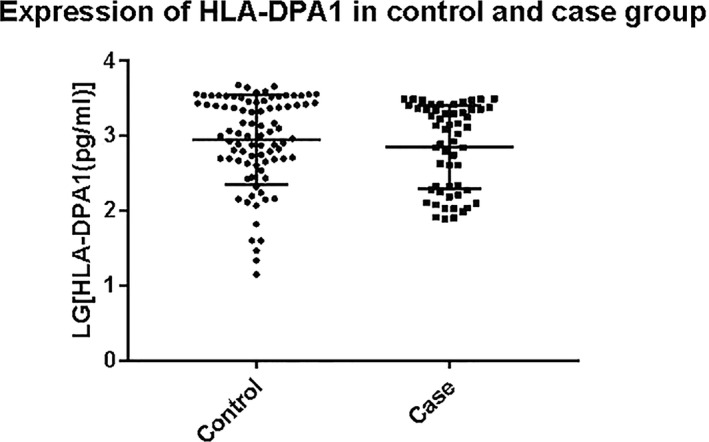
Human leukocyte antigen‐DPA1 (HLA‐DPA1) expression in anembryonic pregnancy and control groups. The ordinate is the concentration of HLA‐DPA1 (GRCh37.p13), which was logarithmically transformed. Case and control group data are presented. *p* = .125, as calculated by the Student's *t* test

**Figure 2:**
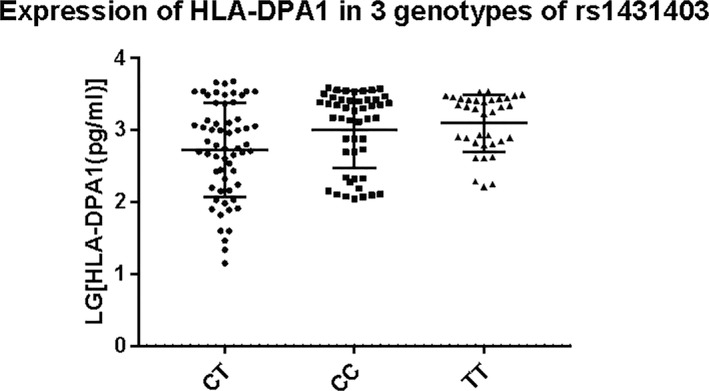
Human leukocyte antigen‐DPA1 (HLA‐DPA1) expression in samples based on rs1431403 genotype. The ordinate is the HLA‐DPA1 (GRCh37.p13) concentration, which was logarithmically transformed. Circular represented samples with their genotypes of the rs1431403 CT, CC, and TT genotypes are presented. *p* = .085, as calculated by analysis of variance

HLA‐DPA1 levels were determined by ELISA in 143 samples of similar gestational week, including 87 controls and 56 cases. The HLA‐DPA1 concentration fluctuated in groups, but the median concentration did not significantly differ between the two groups (control group: 1631.56 ± 1,372.45 pg/ml, case group: 1,295.87 ± 1,086.81 pg/ml, *p* > .05) (Figure 1). The rs1431403 genotypes of all the samples in which HLA‐DPA1 protein expression was measured included 60 CT, 48 CC, and 35 TT. HLA‐DPA1 levels in the CC genotype and TT genotype groups were higher than those in the CT genotype group, but the differences were not significant (*p* > .05) (Figure 2).

### 3.4 Distribution of samples with rs1431403 genotypes among groups with different HLA‐DPA1 levels

To analyze the correlation between rs1431403 genotype and HLA‐DPA1 levels, we separated the samples into subgroups based on HLA‐DPA1 expression levels (100, 500, and 1,500 pg/ml) and assessed the r1431403 genotype frequencies (Table 4). The distribution of samples with CC and TT genotypes in the different HLA‐DPA1 concentration groups significantly differed from that of samples with the CT genotype (*p* < .01). When the concentration of HLA‐DPA1 was below 1,500 pg/ml, the frequency of homozygous samples was lower than that of samples with the CT genotype. An exception to this was samples with the CC genotype in the 100–500 pg/ml group (*n* = 17, 35.4%), and in the ≤100 pg/ml group, which consisted of only 10 samples, all with the CT genotype. In the ≥1,500 pg/ml concentration group, the frequency of homozygous samples (n_CC_ = 21, 43.8%, n_TT_ = 20, 57.1%) was greater than that of heterozygote samples (n_CT_ = 15, 25.0%). These results suggest that rs1431403 polymorphisms might aberrantly activate HLA‐DPA1 expression.

**Table 4:** Expression distribution interval of human leukocyte antigen‐DPA1 (HLA‐DPA1) in different genotypes of rs1431403

rs1431403	HLA‐DPA1 (pg/ml)				*p*
	≦100	100–500	500–1500	≧1,500	
	*N* = 10, *N* (%)	*N* = 49, *N* (%)	*N* = 37, *N* (%)	*N* = 60, *N* (%)	
CT	10 (16.7)	17 (28.3)	18 (30.0)	15 (25.0)	
CC	0	17 (35.4)	10 (20.8)	21 (43.8)	.007
TT	0	6 (17.1)	9 (25.7)	20 (57.1)	.004
CT	10 (16.7)	17 (28.3)	18 (30.0)	15 (25.0)	
CC + TT	0	23 (27.7)	19 (24.9)	41 (49.4)	.000

Note

Chi‐squared tests were used to calculate *p* values. Fisher's exact test was used when the sample size was less than 5.

## 4 DISCUSSION

In this study, the association between *HLA‐DPA1* polymorphisms and risk of anembryonic pregnancy was analyzed in the Chinese Han population. Only one tag SNP, rs1431403, was significantly associated with the risk of anembryonic pregnancy. Homozygous variants of these polymorphic loci, CC and TT, were involved in aberrant increased expression of HLA‐DPA1.

Genotyping results revealed that CC and TT genotypes were identified in 37.5 and 26.6% of case group samples, which was higher than the frequencies observed in the control group (by 16.4 and 10.8% for CC and TT genotypes, respectively). The CT genotype was observed in 35.9% of case group samples, which is 27.3% lower than the frequency at which it was observed in the control group. However, no significant difference in allele frequencies were found between the anembryonic pregnancy group and control group, and the proportion of both C and T alleles were nearly identical. The rs1431403 homozygous genotypes (CC and TT) significantly increased the risk of anembryonic pregnancy. We compared the frequency of rs1431403 genotypes and alleles in the control group with that in other Han populations from Beijing (from the 1,000 Genomes database), and found no significant difference between the two groups (*p* > .05). This result confirmed the accuracy of our typing results and that the frequency of rs1431403 genotypes/alleles in the control group represents those of the Han Chinese population. However, the genotype and allele distributions of rs1431403 in Chinese Han populations significantly differed from those in foreign populations from other Asian countries or other continents, including the Chinese Dai national minority population in Xishuangbanna (*p* < .05). This suggests that the association between rs1431403 and anembryonic pregnancy may differ in different ethnic groups (Table 5).

**Table 5:** Genotype and allele frequencies of rs1431403 polymorphisms in normal populations of various races

Population	*n*	Genotype, *n* (%)			*p*	Allele, *n* (%)		*p*
		CT	CC	TT		C	T	
Chinese (in this study)	114	72 (63.2)	24 (21.1)	18 (15.8)		120 (52.6)	108 (47.4)	
Chinese (Beijing)	103	59 (57.3)	28 (27.2)	16 (15.5)	.560	115 (55.8)	91 (44.2)	0.563
Chinese (Xishuangbanna)	99	31 (31.3)	66 (66.7)	2 (2.0)	.000	163 (82.3)	35 (17.7)	0.000
Japanese (Tokyo)	104	43 (41.3)	37 (35.6)	24 (23.1)	.005	117 (56.3)	91 (43.8)	0.501
European (Northern and Western)	406	177 (43.6)	36 (8.9)	193 (47.5)	.000	249 (30.8)	563 (69.3)	0.000
South American	276	133 (48.2)	45 (16.3)	98 (35.5)	.001	223 (40.4)	329 (59.6)	0.002
North American	337	142 (42.1)	37 (11.0)	158 (46.9)	.000	216 (32.1)	458 (67.9)	0.000
Asian (Southern and Southeastern)	595	252 (42.4)	131 (22.0)	212 (35.6)	.000	514 (43.2)	676 (56.8)	0.009
African	507	232 (45.8)	170 (33.5)	105 (20.7)	.003	572 (56.4)	442 (43.6)	0.303

Note

Polymorphisms in normal populations. Chi‐squared tests were used to calculate *p* values. Fisher's exact test was used when the sample size was less than 5.

Previous studies found that rs1431403 genetic variations exist in the intronic region of *HLA‐DPA1* and *HLA‐DPB1* genes and are involved in autoimmune diseases including rheumatoid arthritis and type 1 diabetes, and in immunodeficiencies such as HIV (Ferreira et al., 2010; Yang, Chang, Liang, Lin, & Wang, 2012). Our study is the first genetic association analysis to explore the relationship between the rs1431403 polymorphism and anembryonic pregnancy in the Chinese Han population. Both HLA Class I and class II molecules play vital roles in presenting antigen peptides (Tsai & Santamaria, 2013). HLA class I molecules are responsible for presenting endogenous peptides. HLA class I molecules combine with endogenous peptides to form a complex which is transported to the cell surface where it is recognized by specific CD8 + T cells and induces cellular immune functions (Andreas et al., 2014). HLA class II molecules are mainly responsible for presenting exogenous antigens. The complex, formed by the combination of HLA II molecules and antigenic polypeptides, is presented at the cell surface where it binds CD4 + T helper lymphocytes (Th) and the T‐cell receptor (TCR). Activation of CD4^+^ Th causes the proliferation of Th cells and expression of the corresponding lymphatic factor, initiating bodily fluid immunity (Goldberg & Rizzo, 2015) (Tineke, Petra, Jongsma, & Jacques, 2011). Increased expression of HLA class II molecules stimulated an increase in the CD4 cells. Ferreira et al. found that the C allele of rs1431403 caused the downregulation of CD4/CD8 both in type 1 diabetes and HIV (Ferreira et al., 2010). In another study, Hsin‐Chou et al. found that variants within the rs1431403 region increased the occurrence of rheumatoid arthritis (RA) (Yang et al., 2012). Upregulation of HLA class II expression is an RA clinical detection index, and some studies have shown that the risk of RA is closely related to HLA genetic polymorphism (Guthrie, Gammill, Madeleine, Dugowson, & Nelson, 2011; Huang et al., 2018; Soumya et al., 2012). Based on these findings, it was inferred that rs1431403 polymorphism increased the incidence of anembryonic pregnancy by inducing abnormal expression of HLA class II molecules, such as HLA‐DP. The results of the current study show that the rs1431403 genotype is associated with HLA‐DPA1 expression. The number of homozygous zygotes with HLA‐DPA1 levels ≥1,500 pg/ml was far higher than that of heterozygous zygotes, while the number of homozygous zygotes with HLA‐DPA1 concentrations <1,500 pg/ml was generally lower than that of heterozygous zygotes. In particular, there was no homozygous zygote in the lowest expression group (≤100 pg/ml). These results validate our hypothesis. While there was no significant difference in HLA‐DPA1 levels between the control and the case groups, combining rs1431403 homozygous genotypes increased the risk of anembryonic pregnancy, and confirmed a quantitative relationship between the rs1431403 genotype, HLA‐DPA1 level, and the risk of anembryonic pregnancy. To some extent, this result explains the possible pathway through which the rs1431403 genotype affects the occurrence of anembryonic pregnancy, but the identification of specific pathological mechanisms requires further studies. Through the prediction of transcription factor binding sites (Alibaba, 2.1, http://gene-regulation.com/pub/programs/alibaba2/index.html), we identified a binding site for interferon consensus sequence binding protein (ICSBP) 4 bp downstream of rs1431403. ICSBP can promote the expression of HLA‐DP (Fabrizio et al., 2006), but further functional studies are required to determine whether *HLA‐DPA1* transcription is affected by the rs1431403 polymorphism.

## 5 CONCLUSION

The rs1431403 polymorphism locus in the *HLA‐DPA1* gene maybe a potential risk factor for anembryonic pregnancy in the Chinese population. This polymorphism may promote anembryonic pregnancy through HLA‐DPA1 upregulation. Our results may provide a new avenue for the detection of anembryonic pregnancy in the first trimester, facilitating an earlier diagnosis of pregnancy loss.

## CONFLICT OF INTEREST

We declare that we have no financial and personal relationships with other people or organizations that can inappropriately influence our work, there is no professional or other personal interest of any nature or kind in any product, service, and/or company that could be construed as influencing the position presented in, or the review of, the manuscript entitled, “Relationship between HLA‐DPA1 Genetic Polymorphism and Anembryonic Pregnancy”.
